# Effects of temperature and superparasitism on quality and characteristics of thelytokous *Wolbachia*-infected *Trichogramma dendrolimi* Matsumura (Hymenoptera: Trichogrammatidae) during mass rearing

**DOI:** 10.1038/s41598-019-54719-5

**Published:** 2019-12-02

**Authors:** Jin-Cheng Zhou, Yuan-Yuan Li, Quan-Quan Liu, Su-Fang Ning, Wu-Nan Che, Bin Cong, Hui Dong

**Affiliations:** College of Plant Protection, Shenyang Agricultural University, No. 120 Dongling Rd, Shengyang, Liaoning 110866 P. R. China

**Keywords:** Ecology, Microbiology, Zoology

## Abstract

Thelytokous *Wolbachia-*infected *Trichogramma* spp. are widely used egg parasitoids against lepidopteran pests in biological control programs. *Wolbachia* may manipulate host wasps for superparasitism and is sensitive to temperature. To explore effects of temperature and superparasitism, we compared fitness parameters and *Wolbachia*-mediated phenotype of thelytokous *Wolbachia*-infected *Trichogramma dendrolimi* between those emerging from superparasitised or single-parasitised hosts at 17, 21, 25, or 29 °C. Infected mothers of *T. dendrolimi* showed reduced superparasitism and parasitism increased with temperature. *Wolbachia* titre decreased with temperature when females emerged from singly-parasitised hosts, but there was no correlation in superparasitised hosts. Females showed higher *Wolbachia* titres at 21, 25, or 29 °C when developing from superparasitised hosts. The daily male ratio of offspring increased with temperature, and the day-age threshold for 5%, 50%, or 95% daily male ratio decreased with temperature in both parasitism forms. Females that emerged from superparasitised hosts had a shorter life span and reduced fecundity. These results indicate that *Wolbachia* may affect host behaviour by increasing superparasitism to enhance its spread, but this has negative effects on thelytokous *Wolbachia*-infected *T. dendrolimi*.

## Introduction

The egg parasitoid wasps *Trichogramma* spp. are the biological control agents against lepidopteran pests in agriculture and forestry^1,2^. Generally, their sex determination mechanism is haplodiploid, wherein males develop from unfertilised haploid eggs and females arise from diploid fertilised eggs^3^. However, some strains of *Trichogramma* can produce nearly 100% females even without mating. This phenomenon is called thelytoky. This form of reproduction in *Trichogramma* is often induced by *Wolbachia* bacteria harboured in its eggs^4–6^, where *Wolbachia* can manipulate chromosome behaviours by gamete duplication during the first mitotic nuclear division^4,7,8^. Thereafter, only females can arise from diploid embryos^9^. Thelytokous *Trichogramma* are often viewed as superior biological control agents since they have a potentially higher rate of reproduction than sexually-reproducing wasps, since all offspring are female, and because host resources are not wasted on males, making them cheaper to produce. Moreover, thelytokous wasps may be easier to establish in fields because released females can produce generations of female offspring without mating^10–12^.

Due to the interaction of *Wolbachia* and their host wasps, the biological characteristics of thelytokous *Wolbachia*-infected *Trichogramma* may be strongly influenced by both abiotic and biotic factors. Temperature can cause variations in *Wolbachia* titres, which then influence *Wolbachia*-mediated phenotype. Reduction of *Wolbachia* titre and/or reduced penetrance of hosts can occur under high^13,14^ or low temperatures^13,15^. In field conditions, thelytokous *Trichogramma* may encounter fluctuating temperature in a day or across seasons, and *Wolbachia* and its host manipulation may be accordingly affected. In addition, host egg resources are often limited in certain seasons. *Trichogramma* females may remain on one host egg mass and lay more eggs in a host, resulting in superparasitism^16–18^, because leaving the egg mass would mean an added risk of failing to find a new one. In superparasitised host eggs, parasitoid offspring may compete for resources and often show delayed development, lower fecundity, smaller body size, and higher mortality^19,20^. Therefore, superparasitism is often viewed as maladaptive for the wasps^21^. However, recent studies have shown that *Wolbachia* can be transmitted both vertically from mother to offspring and horizontally from offspring to offspring when they share the same host egg^22–24^. The potential of horizontal transmission implies a positive effect of superparasitism for the spread of *Wolbachia*. However, little is known about the effects of superparasitism on the interactions between *Wolbachia* and *Trichogramma* and on the effects of temparature.

*Trichogramma dendrolimi* Matsumura is widely used against multiple species of lepidopteran pests in fields across low latitude to high latitude regions^25,26^, making it likely to encounter a wide range of thermal conditions^11,27,28^. *T. dendrolimi* females usually deposit only one egg in a host egg of *Corcyra cephalonica* Stainton (Lepidoptera: Pyralidae) during a single oviposition event, but can also deposit more than one egg during multiple oviposition events, resulting in superparasitism^29,30^. Since determination of superparasitism in *C. cephalonica* is straightforward, we used its eggs in this study.

In this stdy, the female wasps were supplied with *C. cephalonica* eggs at 17 °C, 21 °C, 25 °C, and 29 °C, respectively. We determined single-parasitism and superparasitism rates in host eggs based on the number of offspring per egg. Following this, host eggs were reared until wasp emergence, and the penetrance of *Wolbachia*-mediated parthenogenesis and fitness parameters of *T. dendrolimi* offspring were evaluated at each temperature and parasitism form.

## Results

### Parasitism rate and probability of superparasitism

**Figure 1:**
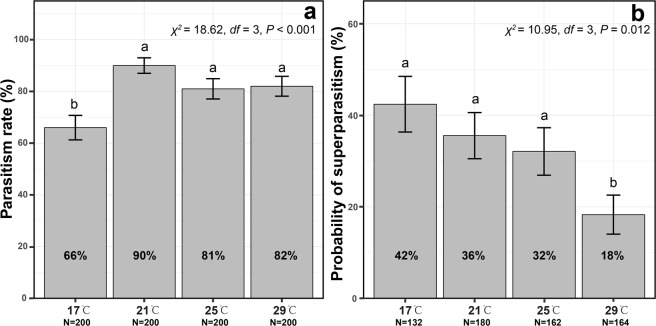
Parasitism rate (**a**) and probability of superparasitism (**b**) as influenced by temperature. The error bars indicate the 95% confidence interval.

Parasitism rates were significantly influenced by temperature (*χ*^2^ = 18.62, *P* < 0.001; Fig. 1a). The parasitism rate at 17 °C (mean = 66.00%) was the lowest, and significantly lower than that at 21 °C (mean = 90.00%, *P* < 0.001), 25 °C (81.00%, *P* = 0.025), or 29 °C (82.00%, *P* = 0.016; Fig. 1a). The probability of superparasitism was also significantly influenced by temperature (*χ*^2^ = 10.95, *P* = 0.54; Fig. 1b). The probability of superparasitism at 29 °C (mean = 18.29%) was the lowest, and significantly lower than that at 21 °C (32.10%, *P* = 0.031), 17 °C (35.56%, *P* = 0.0024), or 25 °C (42.42%, *P* = 0.048; Fig. 1b).

### Daily male ratio, daily incidence rate of intersex, and Wolbachia titre

**Figure 2:**
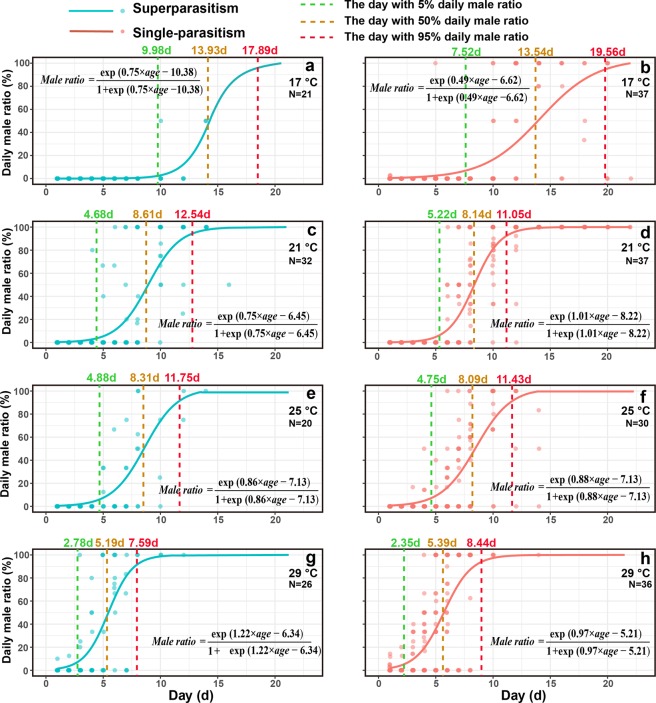
Daily male ratio of offspring as influenced by adult day-age, temperature, and parasitism form.

**Figure 3:**
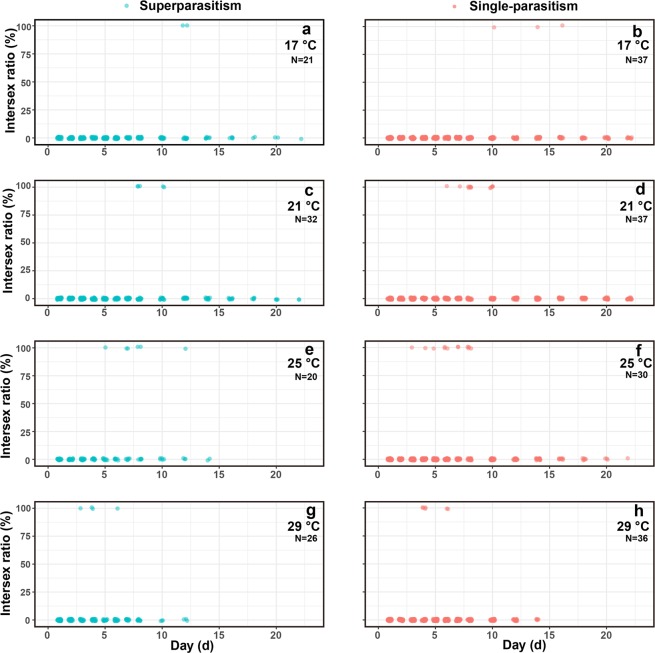
Daily incidence rate of intersex as influenced by adult day-age, temperature, and parasitism form.

Daily male ratio was significantly influenced by the interaction between temperature and day-age of females (*F*_*3*,22*48*_ = 22.41, *P* < 0.001), but was not influenced by parasitism form (*F*_*1*,2*248*_ = 2.38, *P* = 0.12). Although daily male ratio increased with day-age of females at different temperature conditions, the increasing rate at 17 °C was significantly lower than that at 21 °C (z = 6.62, *P* < 0.001), 25 °C (z = 5.41, *P* < 0.001), or 29 °C (z = 7.29, *P* < 0.001), and the increasing rate was not different among 21 °C, 25 °C, or 29 °C (Fig. 2). The day-age threshold for 5%, 50%, and 95% daily male ratio decreased with temperature in both parasitism forms (Fig. 2). Daily incidence rate of intersex was not influenced by temperature (*F*_*3,2250*_ = 2.52, *P* = 0.056), parasitism form (*F*_*1,2248*_ = 0.48, *P* = 0.49), or day-age of females (*F*_*1,2248*_ = 1.99, *P* = 0.16; Fig. 3).

**Figure 4:**
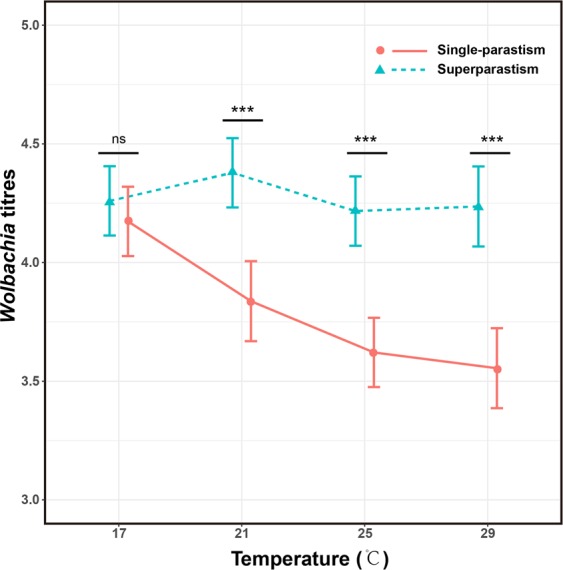
*Wolbachia* titres as influenced by temperature and parasitism form. The error bars indicate the 95% confidence interval.

*Wolbachia* titres of females were significantly influenced by the interaction between parasitism form and temperature (*F*_*1,112*_ = 14.37, *P* < 0.001). Titres significantly decreased (coefficient ± SE: −0.054 ± 0.011, t = 5.12, *P* < 0.001) with temperature when females emerged from single-parasitised hosts, but they were not correlated (−0.0057 ± 0.0069, t = 0.83, *P* = 0.41) with temperature when females emerging from superparasitised hosts (Fig. 4). In addition, *Wolbachia* titres of females emerging from superparasitised hosts were significantly higher than those of females emerged from single-parasitised hosts at 21 °C (z = 5.31, *P* < 0.001), 25 °C (z = 5.86, *P* < 0.001), or 29 °C (z = 6.69, *P* < 0.001), but the difference was not significant at 17 °C (z = 0.86, *P* = 0.39; Fig. 4).

### Fitness parameters of *T. dendrolimi*

**Figure 5:**
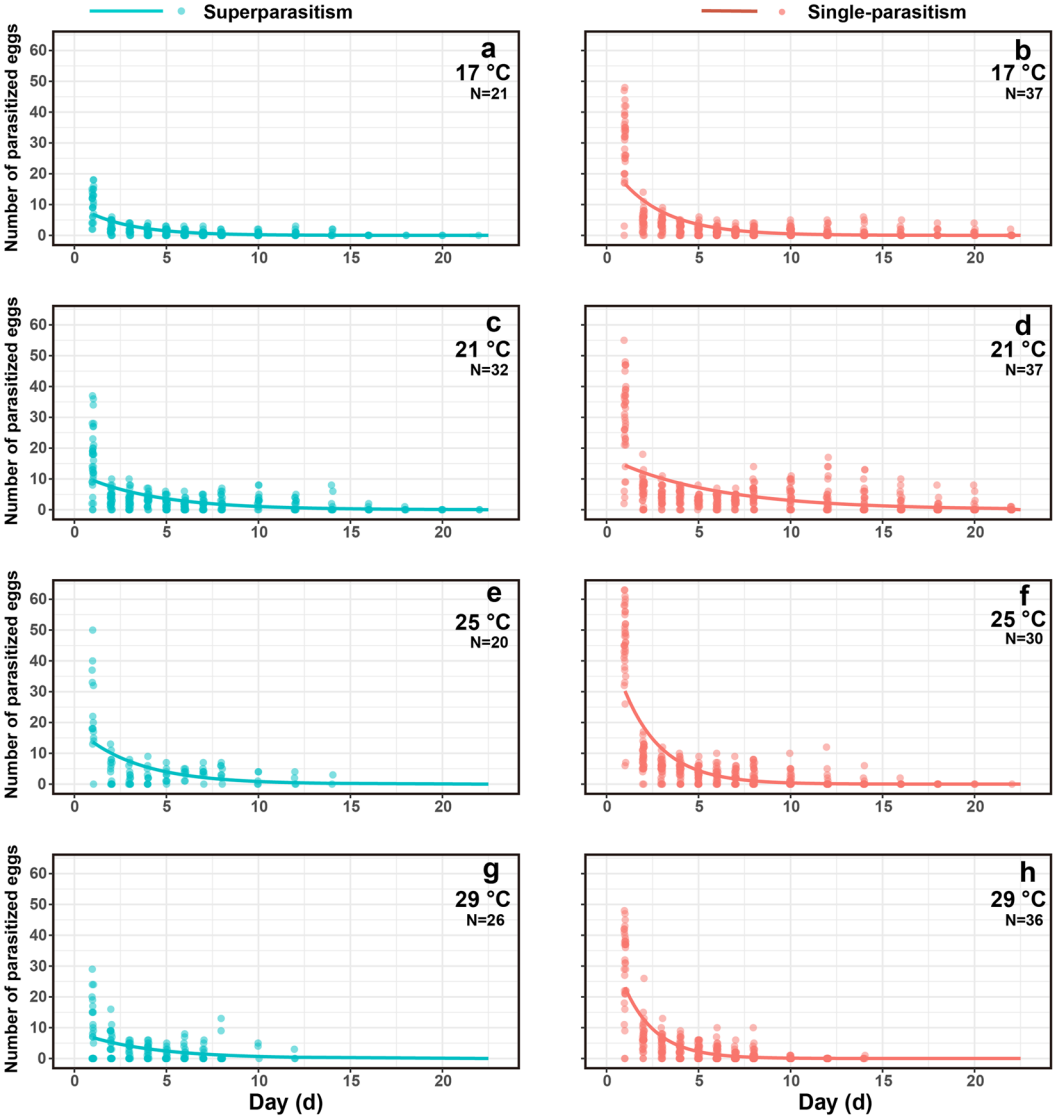
Daily numbers of parasitised eggs as influenced by adult day-age, temperature, and parasitism form.

Daily numbers of parasitised eggs were significantly influenced by day-age of females (*F*_*1,2248*_ = 322.37, *P* < 0.001), temperature (*F*_*3,2248*_ = 169.38, *P* < 0.001), and parasitism form (*F*_*1,2248*_ = 144.49, *P* < 0.001). Daily numbers of parasitised eggs significantly decreased with day-age of females (Coefficient ± SE: −0.44 ± 0.030, *z* = 14.53, *P* < 0.001). We also found that daily numbers of parasitised eggs in single-parasitism form were significantly higher (*z* = 7.33, *P* < 0.001) than those in superparasitism form (Fig. 5).

**Figure 6:**
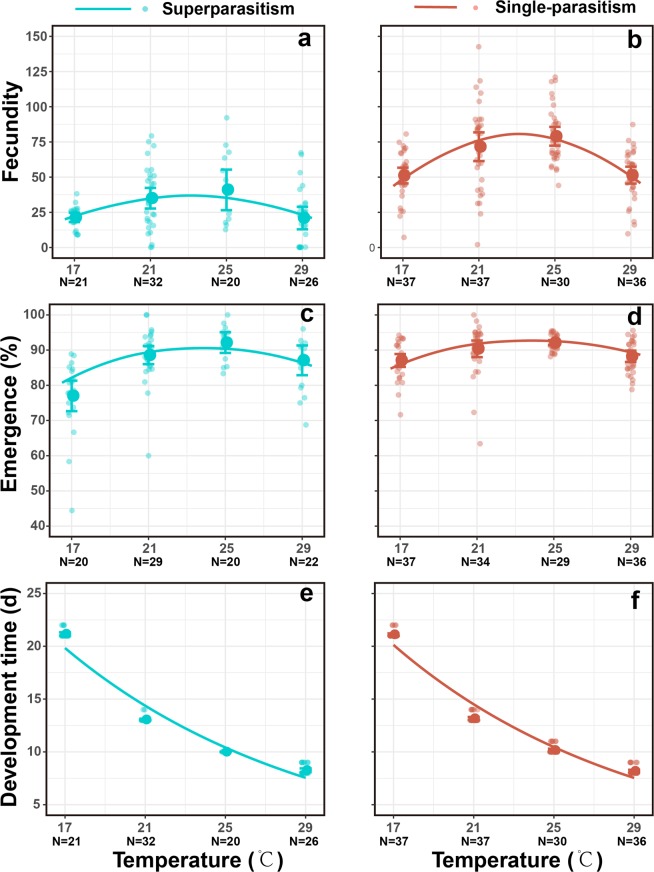
Fecundity, emergence rate of offspring, and development time of females as influenced by temperature and parasitism form. The big solid points indicate the mean values. The small solid points indicate the value of each replicate; The error bars indicate the 95% confidence interval.

Female fecundity was significantly influenced by temperature (*F*_*1,235*_ = 56.02, *P* < 0.001) and parasitism form (*F*_*1,235*_ = 159.01, *P* < 0.001), but not by the interaction of temperature and parasitism form (*F*_*1,235*_ = 1.65, *P* = 0.20). Fecundity of females emerging from single-parasitised hosts (mean ± SE: 80.76 ± 3.56 eggs) was significantly higher (*z* = 11.90, *P* < 0.001) than in females emerging from superparasitised hosts (36.99 ± 3.39 eggs). In addition, female fecundity correlated with increasing temperature (*t* = 7.40, Pseudo R^2^ = 0.46, *P* < 0.001). The optimum temperature for fecundity calculated by GLM regression was 23.12 °C (Fig. 6a,b).

Emergence rate of offspring was significantly influenced by temperature (*F*_*1,223*_ = 53.22, *P* < 0.001) and parasitism form (*F*_*1,223*_ = 12.98, *P* < 0.001), but it was not influenced by interaction of temperature and parasitism form (*F*_*1,223*_ = 1.32, *P* = 0.25). The emergence rate of offspring in single-parasitism (mean ± SE: 82.59 ± 0.35%) was significantly lower (*z* = 4.23, *P* < 0.001) than in superparasitism (90.44 ± 0.57%). Like fecundity, emergence rates of offspring also showed a quadratic correlation with increasing temperature (*z* = 8.46, Pseudo R^2^ = 0.52, *P* < 0.001). The optimum temperature for emergence was 23.87 °C (Fig. 6c,d).

Female development time was not influenced by parasitism form (*F*_*1,237*_ = 0.77, *P* = 0.38) or interaction of parasitism form and temperature (*F*_*1,237*_ = 0.30, *P* = 0.59), but it was significantly influenced by temperature (*F*_*1,237*_ = 4920.98, *P* < 0.001). Development time of females significantly decreased with temperature (Coefficient ± SE: −0.008 ± 0.005, *t* = 15.03, Pseudo R^2^ = 0.93, *P* < 0.001; Fig. 6e,f).

**Figure 7:**
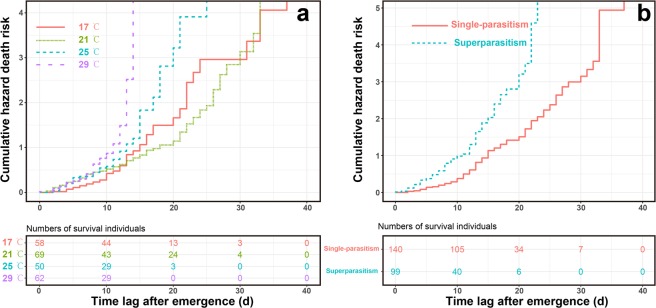
Cumulative hazard death risk and number of surviving individuals as influenced by temperature (**a**) and parasitism form (**b**).

Cox proportional hazard models showed that death risk for female wasps at different day-age was significantly influenced by temperature (*χ*^*2*^ = 55.83, *P* < 0.001) and parasitism form (*χ*^*2*^ = 48.95, *P* < 0.001), but not by interaction of temperature and parasitism form (*χ*^*2*^ = 2.16, *P* = 0.54). Death risk of female wasps emerging from single-parasitised hosts was significantly lower (*z* = 7.17, *P* < 0.001) than in those emerging from superparasitised hosts (Fig. 7b). We also found that death risk of female wasps significantly increased with temperature (*z* = 4.53, *P* < 0.001; Fig. 7a).

## Discussion

*Wolbachia*-infected mothers of *T. dendrolimi* showed a higher parasitism rate and lower proportion of superparasitism at higher temperatures, and *Wolbachia* titres decreased with temperature in normal conditions. Therefore, the effect of temperature on parasitism behaviour might be related to *Wolbachia* titres. As in previous studies^12,31,32^, our results showed that *T. dendrolimi* females declined in fitness when produced by superparasitism, showing shorter life span and reduced fecundity.

Superparasitism is often viewed as maladaptive for wasps due to this reduced fitness^12,21,31,32^. The outcome of the intrinsic competition in superparasitised hosts depends on the usage strategy of different parasitoid species^18^. For solitary parasitoids, which always only allow the survival of a single offspring individual, the parasitoid larvae often destroy competitors through physical attack or physiological suppression. However, a resource-sharing strategy is often observed in gregarious parasitoids, which can allow the survival of more than one offspring individual^18^. *T. dendrolimi* can deposit a clutch of several to hundreds of eggs in a relatively large host egg, but deposits only one egg in a small host egg^29,33,34^. Therefore, this species can be viewed as a facultatively gregarious^35^ and their offspring often survive in the superparasitised host egg though fitness decline. To avoid superparasitism, females can detect and discriminate host quality before depositing eggs and can label the host with host marking pheromone (HMP) after oviposition^21,36^. However, infected females show a lower host discrimination ability^11,23,37^. Because they may be unable to recognise host eggs parasitised by other wasps, they are more likely to lay their own egg clutches, resulting in superparasitism. We also found that females showed a lower rate of superparasitism with lower *Wolbachia* titres induced at high temperature, possibly due to *Wolbachia* inducing memory loss in infected females. Infected wasps often show reduced memory duration, and may “forget” information on host quality or previously parasitised eggs^37^. This hypothesis is supported by studies showing that *Wolbachia* can invade the brain and replicate rapidly in the central nervous system of its host^38,39^. Based on this, we suggest that *Wolbachia* may manipulate host behaviour to enhance its spread. First, if infected females oviposit an egg in a host egg that has been parasitised by uninfected females, this may provide *Wolbachia* an opportunity for transmission from infected to uninfected offspring^24^. Second, it is possible that females remaining and re-parasitizing hosts is a better strategy than leaving the host egg patch^21^. Superparasitism occurs more often when unparasitised hosts are rare (egg limited model) and when short-lived females have many mature eggs (time-limited model)^19,40^, with both outcomes yielding a greater number of offspring. Considering the short life span and limited flight capacity of *Trichogramma* females, they may not prefer to waste progeny eggs when the host resource is limited. In field conditions, when the host egg is limited in certain seasons, infected females can produce more infected offspring despite a decrease in their fitness. Therefore, superparasitism may have several potential positive impacts for use in biological control programs. For instance, total fitness may increase in the superparasitised host as the total number of offspring reaches a maximum, even though the fitness of individual progeny declines^21^. In addition, when host eggs are limited in fields, females save time in searching and handling hosts during their short life span. Females may produce sufficient offsprings through superparasitism^21^, thereby minimizing “wastage” of female eggs and hosts. However, superparasitism may be not a favorable factor for the application of egg parasitoids against pests, as some healthy host eggs will remain in the field, and then hatch. Infection by *Wolbachia* and its lower fitness may appear unfavorable even though all individuals are females, because a non-infected female is expected to destroy more eggs.

Our results also showed increased *Wolbachia* titre in females emerging from superparasitised hosts compared to those emerging from single parasitism. In superparasitised hosts, offspring share and compete for host nutrition due to crowding. Similar increases in *Wolbachia* titre have been found in other host organisms^41^. In contrast, crowding may decrease *Wolbachia* titres^41,42^. For our result, a potential explanation is that the immune response to infection may be energetically costly, with a trade-off existing between immune ability and other aspects of fitness in superparasitism conditions. *Wolbachia* may therefore quickly invade and replicate in the ovary or other tissues of host females in order to minimise immune responses^43,44^. In addition, higher *Wolbachia* titres may increase the probability of horizontal transmission from infected to uninfected host offspring in shared host eggs in superparasitism conditions. This possibility should be further investigated.

We found that the daily male ratio of offspring increased with temperature regardless of whether mothers emerged from singly parasitised or superparasitised hosts, and that the day-age threshold for 5%, 50%, and 95% daily male ratio decreased with temperature in both parasitism forms. Altered male ratios are often linked to variation of *Wolbachia* titres in females, and the reduction of *Wolbachia* density in high temperature has been observed in a number of studies^9,31,45–47^. Stouthamer suggested that reduced *Wolbachia* titre might inhibit the process of gamete duplication during the egg’s first mitotic division^32^, so that an increase in the production of males can be induced by the reduced *Wolbachia* titre as influenced by increasing heat^17,47^.

Interestingly, in superparasitism conditions, though *Wolbachia* titres did not decrease with temperature, the daily male ratio of offspring and the day-age threshold still increased with temperature. Other studies have also shown that reduced *Wolbachia* titres may not always be correlated with *Wolbachia*-mediated phenotypes^9,48^. The final determinant of successful parthenogenesis induction of may be determined by *Wolbachia* titres as well as an unknown factor of *Wolbachia* that needs to be of sufficient level. Zchori-Fein *et al*. hypothesized this mechanism as the sex-ratio changes in a pupal parasitoid wasp, *Muscidifurax* spp^48^. This has been also hypothesized as the mechanism for inducing cytoplasmic incompatibility between sperm and ovum induced by *Wolbachia*, wherein sperm do not contain *Wolbachia*, but are modified by *Wolbachia*-derived proteins^49–51^. As such, disruption of *Wolbachia*-derived protein expression may be induced by environmental stressors. The expression of *Wolbachia*-derived proteins under various conditions should be evaluated in order to help identify a threshold level for successful induction of parthenogenesis in the future.

In this study, we found an increase in the daily male ratio with age of females. A reduction in transmission efficacy and poor host manipulation of *Wolbachia* in older females has been similarly reported in other studies^17,50,52^. Lindsey and Stouthamer suggested that the reproductive rate might mediate the level of male production in thelytokous *Trichogramma*. After long-playing constant oviposition, *Wolbachia* populations could continually be transferred from somatic tissues to the germline to ensure high titres in infected eggs^17^, resulting in lower whole-body titres. By this process, females would produce more males as *Wolbachia* titres decreased with the age of females. Under natural conditions, male production might depend on resources in the altered host^50,53^. When the number of host eggs is limited, wasps are unable to parasitise host eggs continuously. As such, the male ratio may remain at lower levels as access to host eggs is limited. However, when host eggs are constantly available for females, females can quickly find more host eggs in their early adult life, and can lay eggs continuously, leading to the production of more males. To avoid the too high male ratio of *Trichogramma* production in mass rearing, we suggest the duration for parasization of thelytokous *Wolbachia*-infected *Trichogramma* spp should be controlled and limited within five days since females emerged.

Similar to most studies^54–56^, we also found *Trichogramma* offspring achieved maximum fitness at an intermediate temperature and their development rate increased with temperature. Though *Trichogramma* offspring gain maximum fitness at an an intermediate temperature with a relatively low growth rate, the slow growth-high mortality hypothesis (SG-HG) predicts that slower growing larvae suffer greater mortality due to prolonged exposure to natural enemies in field conditions^57,58^. Nevertheless, Bergant & Trdan^59^ argued that the effects of temperature, based on laboratory experiment, may suffer from a great amount of uncertainty in practice. *Trichogramma* wasps are small insects that are easily affected by dramatic temperature fluctuations. In this study, to exclude effects of the thermal background of laboratory conditions, the *T. dendrolimi* populations were divided into four groups and reared at 17 °C, 21 °C, 25 °C, or 29 °C for ten generations, respectively.

In conclusion, our study provides more evidence that host manipulation by *Wolbachia* may affect parasitic behaviour of infected wasps *Wolbachia* may affect host behaviour by increasing superparasitism to enhance its spread, but this has negative effects on thelytokous *T. dendrolimi*. Given the interest in applying *Wolbachia* as a tool to improve the characteristics of the biological control agents against pests, it is critical that we understand the context-dependent nature of *Wolbachia* mediated phenotypes of host insects, and how these results in different selective pressures for the association between *Wolbachia* and their hosts. We believe that these findings have implications for applying thelytokous *T. dendrolimi* for pest control in various geographical locations.

## Material and Methods

### Insects

The thelytokous isofemale line of *T. dendrolimi* and its hosts *C. cephalonica* were maintained by the Pest Biological Control Laboratory at Shenyang Agricultural University. Both species were reared in glass tubes (diameter, 12 mm; height, 100 mm) closed by a cotton plug under 24 ± 1 °C, 70 ± 5% RH, and 16:8 h light/dark cycles.

The thelytokous isofemale line of *T. dendrolimi* was originally obtained from an egg of *Dictyoploca japonica* Butler (Lepidoptera: Saturniidae) collected from walnut trees (*Juglans regia* L.) in a forest located in Huairen County, Liaoning Province, China (41°23′N, 125°36′E; Altitude: 400.73 m), in May 2015. Thereafter, the females were reared in laboratory conditions for at least 60 generations. Infection by *Wolbachia* in females was detected by using forward primer 5′-TGGTCCAATAAGTGATGAAGAAAC-3′ and reverse primer 5′-AAAAATTAAACGCTACTCCA-3′ for the *wsp* gene of PI-*Wolbachia* strains^11^. In all generations, females produced nearly 100% female offspring even without mating. Vertical transmission of *Wolbachia* from mothers to offspring was stable in all generations.

*C. cephalonica* hosts were reared on a semi-artificial diet. Host eggs were collected in groups of ca. 300 and glued onto a 10 × 10 cm white card with gum arabic for experimental use.

### Experimental procedure

**Figure 8:**
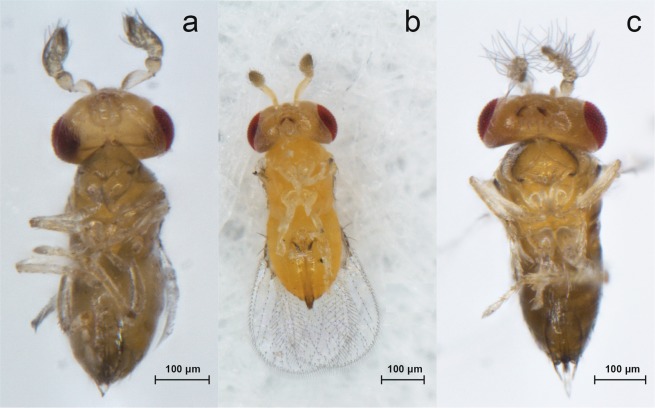
Secondary sexual characteristics of intersex (**a**), female (**b**), and male (s) individuals.

To exclude potential effects of the thermal background of laboratory conditions at 24 °C, the infected *T. dendrolimi* population was divided into four groups and reared at 17 °C, 21 °C, 25 °C, or 29 °C in glass tubes for ten generations. To avoid the effect of superparasitism on mothers, females singly emerged from a host egg were used as mothers. To obtain a sufficient number of superparasitised host eggs, a group of 50 females was supplied with a host egg card with 200 fresh host eggs for 4 h at each temperature treatment, after which the wasps were removed. The host eggs were reared until eggs blackened, which indicated the prepupal stage of *Trichogramma* offspring; failing to blacken indicated unsuccessful parasitism. The blackened host eggs were cut off gentlely by the graver and transferred singly into a small Durham glass tube (diameter, 6 mm; length, 30 mm) by the pincette, then reared individually until wasps emerged under 24 ± 1 °C, 70 ± 5% RH, and 16:8 h light/dark cycles. After emergence, blackened eggs were dissected under a stereozoom microscope (Olympus, SZX16) to confirm whether eggs contained other *Trichogramma* offsprings or not. Based on this, parasitism forms were classified as either single-parasitism or superparasitism, where single-parasitism was defined as an egg containing only one *T. dendrolimi* offspring, and superparasitism was defined as an egg containing at least two *T. dendrolimi* offsprings. The numbers of superparasitised and single-parasitised host eggs were recorded. *T. dendrolimi* development time was defined as the time from oviposition to adult emergence. As offspring emerged, females were reared singly in Durham glass tubes (6 mm diameter, 30 mm length) and supplied with a 20% honey solution via a small cotton ball daily. Every female wasp was supplied with a new host egg card with 300 eggs daily until death. Total 239 females were examined in this study. Lifespans of *T. dendrolimi* wasps were recorded in days. The daily egg cards were reared singly in glass tubes until the emergence of offspring. Daily numbers of parasitised host eggs and daily emergence rates of *T. dendrolimi* offspring were recorded. After the emergence of offspring, sexes of offspring adults were detected based on tentacle characteristics (Fig. 8b,c). Daily male ratios in host egg cards were recorded and intersex individuals of *T. dendrolimi* offspring were identified^9^ (Fig. 8a). Due to the rare frequency of intersex in an egg card, the occurrence of intersex was recorded as binomial data, where 1 represented the occurrence of intersex and 0 represented the non-occurrence of intersex.

### Quantification of wolbachia titres

To determine the *Wolbachia* titre of female individuals under different temperatures in the two parasitism forms, a group of sixteen female replicates was used in each treatment. Females that emerged within 24 h were used in the experiment.

Absolute quantitative PCR (AQ-PCR) was used to measure *Wolbachia* titres. Total DNA was extracted from a single female individual using Chelex-100^8,17^. Specific primers used to determine *Wolbachia* titres of females were designed based on the sequences of *Wolbachia* surface protein (*wsp*) gene (GenBank Accession: MG914000), which is a single copy gene, by Primer 5.0 software and DNAMAN software (forward: 5′-ATGATGTAGCCCCAGAAAT-3′ and reverse: 5′-CACCAAAAGTGTTGTAAAGAA-3′)^60^. A 20-µl qPCR was performed containing 0.5 µl of each primer, 1 µl DNA, 10 µl SYBR Premix Ex TaqTMII (Promega, USA), and 8 µl double-distilled water. A CFX96TM thermocycler (Bio-Rad, USA) was used with the following procedure: 95 °C for 3 min, followed by 40 cycles of 60 °C for 30 s, 65 °C for 5 s, and thereafter increasing 0.5 °C per 5 s to 95 °C. To ensure specificity of amplification, melting curves were generated at the end of each reaction. A standard curve was generated based on the cycle threshold (Ct) reflecting the concentration of the wsp gene, and calculated using a regression equation based on the log-transformed value of the copy number of the wsp gene in different concentrations. Copy numbers of the wsp gene in *T. dendrolimi* females were determined using the equation

1 $$Y=-3.502X+41.890$$

where, Y represents Ct and X represents the log-transformed value of the copy number of the *wsp* gene.

### Data analysis

Post-hoc pairwise comparisons were made using chi-square tests to examine the probability of superparasitism and the parasitism rate as influenced by temperature. Generalised mixed effects models (GLMMs) were applied to determine the effect of day-age of females, temperature, and parasitism form on daily numbers of parasitised eggs, daily male ratio of offspring, daily incidence rate of intersex, and *Wolbachia* titres. To exclude random error from female individuals and their host eggs, the identity of females and their hosts were set as random factors in GLMMs. Based on previous studies and our results showing that the daily male ratio of offspring increased with day-age of females^17^, we calculated the average day-ages of females with 5%, 50%, and 95% male ratio, which respectively indicated the threshold of day-ages that females can be described as beginning to, partly, or nearly completely losing the parthenogenetic phenotype.

*Wolbachia* titres, development time, and fecundity of females and emergence rates of offspring as influenced by temperature and parasitism form were analysed using generalised mixed effects models (GLMs). Cox’s proportional hazard model was applied to quantify the death risks of female wasps at different day-age as influenced by temperature and parasitism form^61^.

All analyses were carried out using R ver. 3.4^62^. The GLMMs were realized by “lme4” package. The Cox’s proportional hazard models were carried by “survival”package^63^. Homogeneities of GLMMs, GLMs, and Cox’s proportional hazard models were tested using studentised Breusch-Pagan tests and Shapiro tests realized by “lmtest” package^64,65^.

## References

[1] Smith SM (1996). Biological control with *Trichogramma*: advances, successes, and potential of their use. Annu. Rev. Entomol..

[2] Li, L. Y. Worldwide use of *Trichogramma* for biological control on different crops: a survey. Biological Control with Egg Parasitoids. (eds. Wajnberg, E. & Hassan, S. A.), 37–53 (CAB, Wallingford, 1994).

[3] Cook JM (1993). Sex determination in the Hymenoptera: A review of models and evidence. Heredity.

[4] Werren JH, Baldo L, Clark ME (2008). *Wolbachia*: master manipulators of invertebrate biology. Nat. Rev. Microbiol..

[5] Zug R, Hammerstein P (2012). Still a host of hosts for *Wolbachia*: analysis of recent data suggests that 40% of terrestrial arthropod species are infected. PLoS One.

[6] Russell JE, Stouthamer R (2011). The genetics and evolution of obligate reproductive parasitism in *Trichogramma pretiosum* infected with parthenogenesis-inducing *Wolbachia*. Heredity.

[7] Tulgetske GM, Stouthamer R (2012). Characterization of intersex production in *Trichogramma kaykai* infected with parthenogenesis-inducing *Wolbachia*. Naturwissenschaften.

[8] Stouthamer R, Breeuwer JA, Hurst GD (1999). *Wolbachia pipientis*: microbial manipulator of arthropod reproduction. Annu. Rev. Microbiol..

[9] Stouthamer R, Luck RF, Hamilton WD (1990). Antibiotics cause parthenogenetic *Trichogramma* (Hymenoptera: Trichogrammatidae) to revert to sex. PNAS.

[10] Pannebakker BA, Zwaan BJ, Beukeboom LW, Alphen JJM (2004). Genetic diversity and *Wolbachia* infection of the *Drosophila* parasitoid *Leptopilina clavipes* in western Europe. Mol. Ecol..

[11] Liu QQ (2018). Decision-making in a bisexual line and a thelytokous *Wolbachia*-infected line of *Trichogramma dendrolimi* Matsumura (Hymenoptera: Trichogrammatidae) toward their hosts. Pest Manag. Sci..

[12] Stouthamer R, Luck RF (1993). Influence of microbe-associated parthenogenesis on the fecundity of *Trichogramma deion* and *T. pretiosum*. Entomol. Exp. Appl..

[13] Bordenstein SR, Bordenstein SR (2011). Temperature affects the tripartite interactions between bacteriophage WO, *Wolbachia*, and cytoplasmic incompatibility. PLoS One.

[14] Hurst LD, Schulenburg JH, Fuyama Y, Johnson PRA (2000). Male-killing *Wolbachia* in *Drosophila*: a temperature-sensitive trait with a threshold bacterial density. Genetics.

[15] Enigl M, Zchori-Fein E, Schausberger P (2005). Negative evidence of *Wolbachia* in the predaceous mite *Phytoseiulus persimilis*. Exp. Appl. Acarol..

[16] Kleber DSP, Guedes NMP, Serrão JE, Zanuncio JC, Guedes RNC (2017). Superparasitism, immune response and optimum progeny yield in the gregarious parasitoid *Palmistichus elaeisis*. Pest Manag. Sci..

[17] Lindsey ARI, Stouthamer R (2017). Penetrance of symbiont-mediated parthenogenesis is driven by reproductive rate in a parasitoid wasp. Peerj.

[18] Harvey JA, Poelman EH, Tanaka T (2013). Intrinsic inter- and intraspecific competition in parasitoid wasps. Annu. Rev. Entomol..

[19] Tunca H, Buradino M, Colombel EA, Tabone E (2016). Tendency and consequences of superparasitism for the parasitoid *Ooencyrtus pityocampae* (Hymenoptera: Encyrtidae) in parasitizing a new laboratory host, *Philosamia ricini* (Lepidoptera: Saturniidae). Eur. J. Entomol..

[20] Devescovi F (2017). Effects of superparasitism on immature and adult stages of *Diachasmimorpha longicaudata* Ashmead (Hymenoptera: Braconidae) reared on *Ceratitis capitata* Wiedemann (Diptera: Tephritidae). Bull. Entomol. Res..

[21] Van Alphen JJM, Visser ME (1990). Superparasitism as an adaptive strategy for insect parasitoids. Annu. Rev. Entomol..

[22] Parratt SR (2016). Superparasitism Drives Heritable Symbiont Epidemiology and Host Sex Ratio in a Wasp. PLoS Pathog..

[23] Farahani KH (2015). Does *Wolbachia* infection affect decision‐making in a parasitic wasp?. Entomol. Exp. Appl..

[24] Huigens ME, de Almeida RP, Boons PA, Luck RF, Stouthamer R (2004). Natural interspecific and intraspecific horizontal transfer of parthenogenesis-inducing *Wolbachia* in *Trichogramma* wasps. Proc. Biol. Sci..

[25] Huang J, Hua HQ, Zhang F, Li YX (2017). Suitability assessment of three *Trichogramma* species in the control of *Mythimna separata* (Lepidoptera: Noctuidae). J. Appl. Entomol..

[26] Dong H, Liu Q, Xie L, Cong B, Wang H (2017). Functional response of *Wolbachia*-infected and uninfected *Trichogramma dendrolimi* Matsumura (Hymenoptera: Trichogrammatidae) to Asian corn borer, *Ostrinia furnacalis* Guenée (Lepidoptera: Pyralidae) eggs. J. Asia-Pac. Entomol..

[27] Xin L, Han S, Li Z, Li L (2017). Biological characters of *Trichogramma dendrolimi* (Hymenoptera: Trichogrammatidae) reared *in vitro* versus *in vivo* for thirty generations. Sci. Rep..

[28] Zhao S, Chen D (1993). A study of hybridization in reproductive characters of five populations in *Trichogramma dendrolimi*. J. Huazhong Agricultural Univ..

[29] Wang L, Huang J, Dong X, Zhang F, Li YX (2015). Superparasitism and ontogeny of two *Trichogramma* species on *Corcyra cephalonica* (Stainton). Chin. J. Biol. Control.

[30] Li YX, Dai GH, Fu WJ (2008). Suitability of *Corcyra cephalonica* to three *Trichogramma* species and change of the content of free amino acids in its eggs parasitized. Acta Entomol. Sin..

[31] Stouthamer R (1993). The use of sexual versus asexual wasps in biological control. Entomophaga..

[32] Hohmann CL, Luck RF, Stouthamer R (2001). Effect of *Wolbachia* on the survival and reproduction of *Trichogramma kaykai* Pinto & Stouthamer (Hymenoptera: Trichogrammatidae). Neotrop. Entomol..

[33] Kong J, Peng H, Chen HY, Bao JZ (1988). Unusual mating behaviour of *Trichogramma dendrolimi* (Hym. Trichogrammatidae) reared on oak silkworm eggs. Chin. J. Biol. Control..

[34] Takada Y, Kawamura S, Tanaka T (2001). Host preference of *Trichogramma dendrolimi* (Hymenoptera: Trichogrammatidae) on its native host, *Mamestra brassicae* (Lepidoptera: Noctuidae) after 12 continuous generations on a factitious host. Appl. Entomol. Zool..

[35] Martel V, Boivin G (2010). Unequal distribution of local mating opportunities in an egg parasitoid. Ecol. Entomol..

[36] Van Dijken MJ, Waage JK (1987). Self and conspecific superparasitism by the egg parasitoid *Trichogramma evanescens*. Entomol. Exp. Appl..

[37] Farahani HK (2017). Decrease of memory retention in a parasitic wasp: an effect of host manipulation by *Wolbachia*?. Insect Sci..

[38] Strunov A, Kiseleva E, Gottlieb Y (2013). Spatial and temporal distribution of pathogenic *Wolbachia* strain wmelpop in *Drosophila melanogaster* central nervous system under different temperature conditions. J. Invertebr. Pathol..

[39] Strunov A, Kiseleva E (2016). *Drosophila melanogaster* brain invasion: pathogenic *Wolbachia* in central nervous system of the fly. Insect Sci..

[40] Godfray, H. C. J. *Parasitoids: behavioral and evolutionary ecology*. (eds Krebs, J. R. & Clutton-Vrock, T.). 126–149 (Princeton University Press, 1994).

[41] Dutton TJ, Sinkins SP (2004). Strain-specific quantification of *Wolbachia* density in *Aedes albopictus* and effects of larval rearing conditions. Insect Mol. Biol..

[42] Wiwatanaratanabutr I, Kittayapong P (2009). Effects of crowding and temperature on *Wolbachia* infection density among life cycle stages of *Aedes albopictus*. J. Invertebr. Pathol..

[43] Wiwatanaratanabutr I, Grandjean F (2016). Impacts of temperature and crowding on sex ratio, fecundity and Wolbachia infection intensity in the copepod *Mesocyclops thermocyclopoides*. J. Invertebr. Pathol..

[44] McKean KA, Nunney L (2001). Increased sexual activity reduces male immune function in *Drosophila melanogaster*. PNAS.

[45] Xi C (2016). Temperature regulates the reproduction mode of *Trichogramma embryophagum* (Hymenoptera: Trichogrammatidae) by influencing the titer of endosymbiont *Wolbachia*. Acta Entomol. Sin.

[46] Zhu LY (2012). *Wolbachia* strengthens *Cardinium*-induced cytoplasmic incompatibility in the spider mite *Tetranychus piercei* McGregor. Curr. Microbiol.

[47] Ross PA (2017). Wolbachia infections in *Aedes aegypti* differ markedly in their response to cyclical heat stress. PLoS Pathogens.

[48] Zchori-Fein E, Gottlieb Y, Coll M (2000). *Wolbachia* density and host fitness components in *Muscidifurax uniraptor* (Hymenoptera: Pteromalidae). J. Invertebr Pathol..

[49] Beckmann JF, Ronau JA, Hochstrasser MA (2017). *Wolbachia* deubiquitylating enzyme induces cytoplasmic incompatibility. Nature Microbiol.

[50] Hohmann CL, Luck RF, Stouthamer R (2001). Host deprivation effect on reproduction and survival of *Wolbachia*-infected and uninfected *Trichogramma kaykai* Pinto & Stouthamer (Hymenoptera: Trichogrammatidae). Neotropical Entomol.

[51] Lepage DP (2017). Prophage wo genes recapitulate and enhance *Wolbachia*-induced cytoplasmic incompatibility. Nature.

[52] Legner E (1985). Natural and induced sex ratio changes in populations of thelytokous *Muscidifurax uniraptor* (Hymenoptera: Pteromalidae). Ann. Entomol. Soc. Am..

[53] Roy DB, Rothery P, Moss D, Pollard E, Thomas J (2001). Butterfly numbers and weather: predicting historical trends in abundance and the future effects of climate change. J. Anim. Ecol..

[54] Wang B, Ferro DN, Wu J, Wang S (2004). Temperature-Dependent development and oviposition behavior of *Trichogramma ostriniae* (Hymenoptera: Trichogrammatidae), a potential biological control agent for the European Corn Borer (Lepidoptera: Crambidae). Environ. Entomol..

[55] Haile AT (2002). Temperature-dependent development of four egg parasitoid *Trichogramma* species (Hymenoptera: Trichogrammatidae). Biocontrol Sci. Techn..

[56] Krechemer FS, Foerster LA (2015). Temperature effects on the development and reproduction of three *Trichogramma* (Hymenoptera: Trichogrammatidae) species reared on *Trichoplusia ni* (Lepidoptera: Noctuidae) eggs. J. Insect Sci..

[57] Clancy KM, Price PW (1987). Rapid herbivore growth enhances enemy attack: Sublethal plant defenses remain a paradox. Ecology..

[58] Chen KW, Chen Y (2016). Slow-growth high-mortality: A meta-analysis for insects. Insect Sci..

[59] Bergant K, Trdan S (2006). How reliable are thermal constants for insect development when estimated from laboratory experiments?. Entomol Exp Appl..

[60] Braig HR, Zhou W, Dobson SL, O’Neill SL (1998). Cloning and characterization of a gene encoding the major surface protein of the bacterial endosymbiont *Wolbachia pipientis*. J. bacteriol.

[61] Cox DR (1972). Regression Models and Life-Tables. J. Royal. Stat. Soc. Series B..

[62] R Developmental Core Team. R: A language and environment for statistical computing. R Foundation for Statistical Computing, Vienna, Austria. https://www.R-project.org/ (2017).

[63] Faraway J. J. Extending the linear model with R. (CRC Press, Boca Rotan, FL, 2006).

[64] Hall AD (1992). A study of various score test statistics for heteroscedasticity in the general linear model. Math. Comput. Simul..

[65] Koenker R (1981). A note on studentizing a test for heteroscedasticity. J. Econom..

